# Croup in Its Relations to Tracheotomy

**Published:** 1875-03

**Authors:** 


### Books Reviewed.

*Croup in its Relations to Tracheotomy.* By J. Solis Cohen, M. D., etc. Philadelphia: Lindsay & Blakiston, 1874.

This monograph forms a valuable contribution to the subject of croup, and more especially is it valuable from the fact that it discusses the subject of tracheotomy in its relation to the various forms of the disease. The author arrives at the following conclusions :

1. That there are no insuperable contra-indications to tracheotomy in croup.

2. That the administration of an anæsthetic for the purpose of controlling the child's movements is admissible in performing the operation; but that it should be used with great caution.

3. That a careful dissection should be made down to the wind-pipe and hemorrhage be arrested before incising it, whenever there is at all time to do so.

4. That the incision should be made into the trachea as near the cricoid cartilage as possible, to avoid excessive hemorrhage, and subsequent accidents which might occasion emphysema.

5. That a dilator should be used, or a piece of the trachea be excised, whenever any difficulty is encountered in introducing the tube.

6. That the tube should be dispensed with as soon as possible; or altogether if the case will admit of it.

7. That assiduous attention should be bestowed upon the after treatment, especially that of the wound; and a skilled attendant should be within a moment's call for the first twenty-four or forty-eight hours immediately following the operation.

Dr. Cohen's labor in collecting the large number of statistics which this work contains and his conscientious review of the whole subject will be appreciated by every member of the profession who is fortunate enough to possess a copy of the book.

